# Extraneural Metastases From a High-Grade Glioma (HGG) With an H3F3A G34R Mutation

**DOI:** 10.3389/fonc.2019.00373

**Published:** 2019-05-08

**Authors:** Amit Jethanandani, Maria K. Gule-Monroe, Melissa Chen, Jason M. Johnson

**Affiliations:** ^1^Department of Radiation Oncology, MD Anderson Cancer Center, Houston, TX, United States; ^2^University of Tennessee Health Science Center, College of Medicine, Memphis, TN, United States; ^3^Department of Diagnostic Radiology, University of Texas MD Anderson Cancer Center, Houston, TX, United States

**Keywords:** glioma, metastasis, spine, MRI, CNS

## Abstract

Distant metastatic disease from gliomas is extremely rare. We report the case of a 17-year-old female with an H3F3A G34R mutated infiltrative glioma who developed painful osseous metastases to her pelvis and spine within 3 months of clinical presentation. The presence of an H3F3A mutation in these patients may indicate further work-up to include intensive staging examination.

## Background

Pediatric diffuse gliomas are a spectrum of primary central nervous system (CNS) tumors distinguished from their adult counterparts by morphologic and phenotypic characteristics as well as prognostic, genotypic markers (1). Within this category, pediatric high-grade gliomas (HGGs) demonstrate multi-modality treatment resistance and consequently poor median survival (2). Although CNS tumors represent the second most common cancer diagnosis in pediatric populations, HGGs account for only 8–12% of incident cases (3). Distant metastasis (DM) of HGG is extremely rare, likely attributable to limited routes of extraneural spread and short duration of patient survival (4). However, case reports suggest HGGs, and glioblastomas (GBMs) in particular, can demonstrate extracranial escape through vascular or perineurial invasion as well as lymphatic or iatrogenic spread, assuming patients exceed thresholds of expected survival (4). Despite the biologic plausibility of DM in these cases, data remain sparse, and a precise mechanism has yet to be elucidated.

Some biological subgroups of pediatric HGGs, characterized by somatic histone mutations (e.g., H3F3A K27M, H3F3A G34R/V, and HIST1H3B/C), underlie distinct profiles of gliomagenesis (5). In a recent report of two pediatric HGG patients with osseous metastases, the only overlapping mutations identified via whole-exome sequencing were H3F3A K27M mutations. Neither patient had osseous metastasis on initial presentation, which subsequently developed at 3.5 months and 5.5 months, respectively, suggesting an aggressive tumor biology (6).

We describe a case of pediatric HGG with an H3F3A G34R mutation and extraneural metastases. HGGs with G34 mutations are typically diagnosed in adolescence or young adulthood (age range: 10–25 years). They commonly arise in hemispheric or cortical structures and present along with a gradient of GBM, GBM-like, PNET-like, and PNET morphology. Median survival is estimated to be 22 months, possibly as a consequence of the high frequency of MGMT promoter methylation in these tumors and subsequent favorable response to chemotherapy protocols (4, 6).

## Case

**Figure 1:**
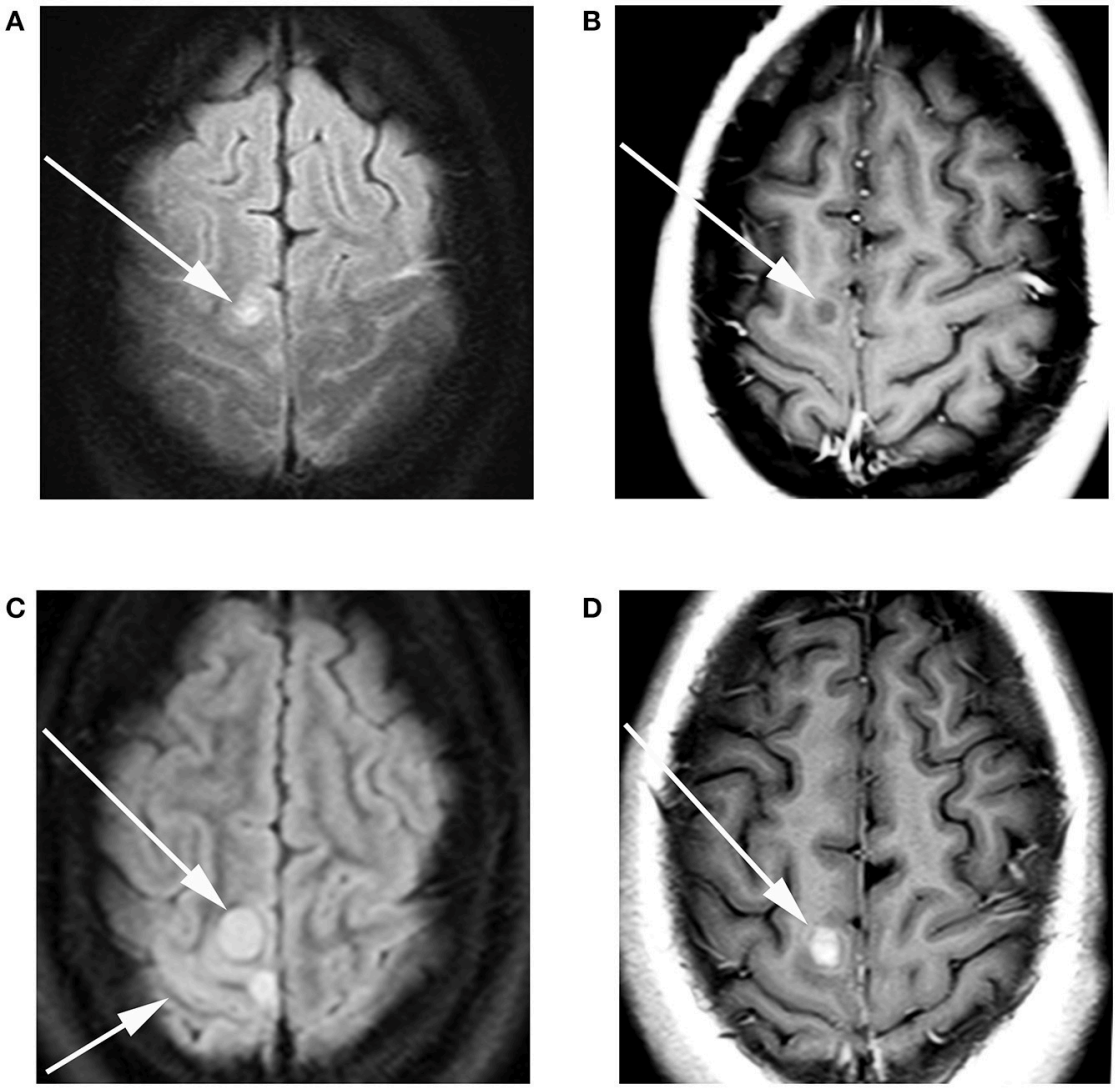
A 17-year-old female with H3F3A G34R mutated infiltrative glioma. MRI of the brain with and without contrast at the time of presentation revealed **(A)** a T2 FLAIR hyperintense focus (arrow) with adjacent T2 FLAIR hyperintense cortical thickening. **(B)** T1 contrast-enhanced imaging revealed faint peripheral enhancement (arrow). Three-month follow-up imaging revealed **(C)** increase in the size of the T2 FLAIR hyperintense lesion long arrow with associated cortical thickening short arrow. **(D)** T1 contrast-enhanced imaging revealed a solid enhancing nodule (arrow).

**Figure 2:**
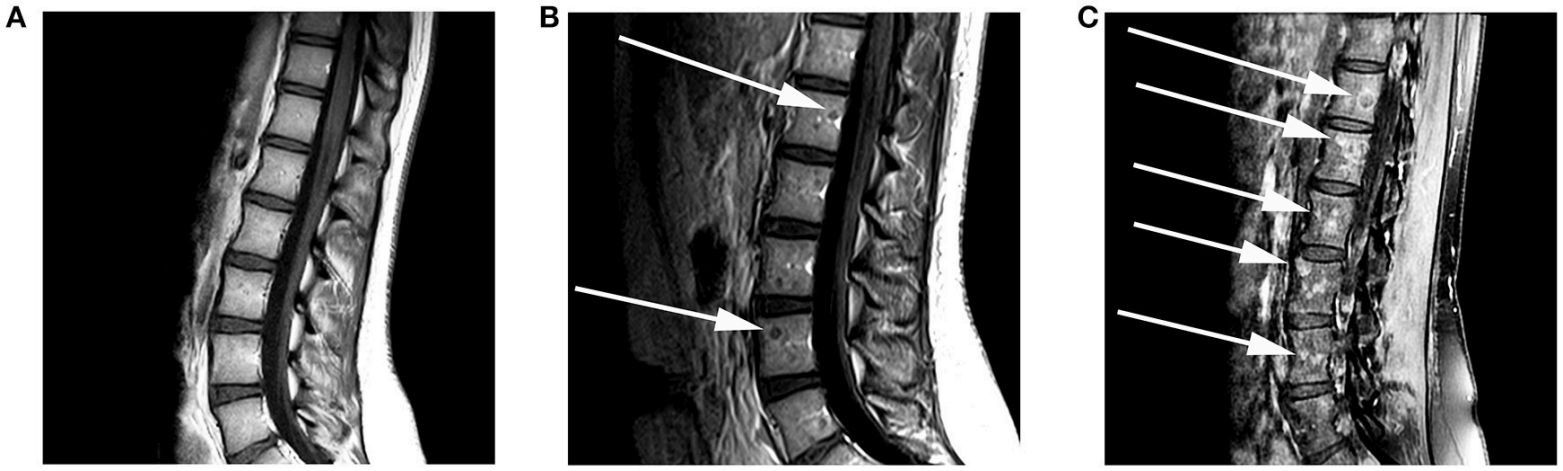
A 17-year-old female with H3F3A G34R mutated infiltrative glioma. MRI of the spine with and without contrast at the time of presentation was performed. **(A)** A sagittal T1 pre-contrast image of the lumbar spine shows no evidence of a marrow replacing lesion. **(B)** Follow-up imaging 3 months later reveals the development of rim-enhancing marrow replacing lesions (arrows) within the lumbar vertebral bodies on this sagittal T1 contrast-enhanced image. **(C)** Five months later there was a significant increase in the size and number of enhancing marrow replacing lesions (arrows) on this sagittal T1-post contrast fat saturated image.

**Figure 3:**
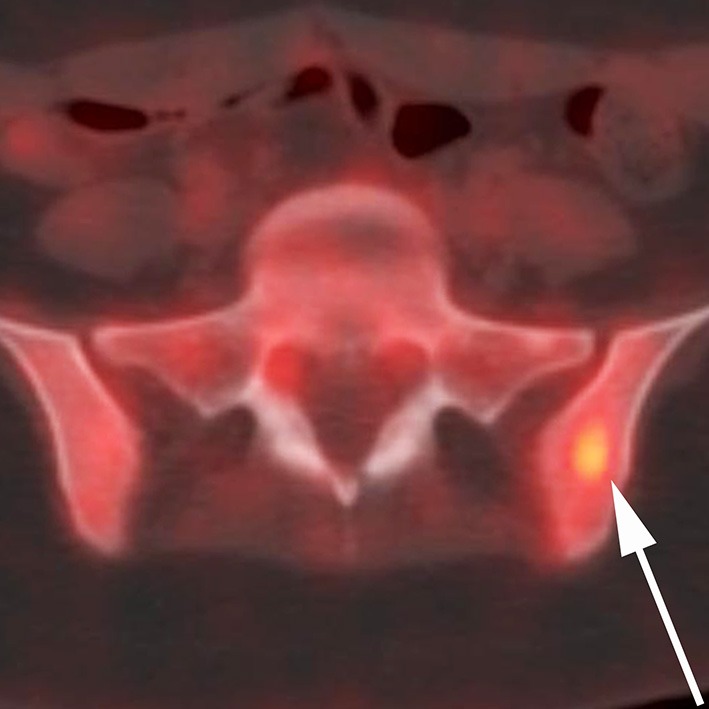
A 17-year-old female with H3F3A G34R mutated infiltrative glioma. 18F-FDG PET CT fused axial image of the superior bony pelvis reveals a hypermetabolic sclerotic lesion (maximum SUV of >6) within the left iliac bone (arrow).

Patient consent for publication was obtained. A 17-year old female presented to an outside hospital (OSH) after she fell down the stairs in her home. She attributed the fall to a 1-month history of numbness, tingling, and weakness in her bilateral lower extremities (BLE), back pain, as well as the sudden onset of blurry vision. On physical exam, she was found to be areflexic in both legs. An initial non-contrast MRI of the brain shortly after admission (Figure 1A) revealed a subcentimeter focus of subcortical and cortical T2 hyperintensity in the right paramedian motor cortex. This lesion faintly peripherally enhanced (Figure 1B). This was initially thought to be unrelated to her symptoms. A contrast-enhanced MRI of the spine was unremarkable at this time (Figure 2A). The patient was diagnosed with Guillain-Barre Syndrome and treated with high-dose steroids. Her symptoms did not improve, and the treatment regimen was switched to intravenous immunoglobulin (IVIG). She completed 5 days of IVIG with minimal improvement and was discharged to an inpatient rehabilitation facility. Unfortunately, the patient did not respond to physical therapy (PT), and, in the interim, developed daily headaches associated with photophobia, phonophobia, diplopia, and nausea. No anti-neoplastic treatment was provided. During her PT, she presented to an OSH Emergency Room (ER) with new right arm weakness and slurred speech. Contrast-enhanced MRIs of the brain revealed the progression of her right paramedian motor cortex lesions which had increased in size with solid central enhancement seen of the larger lesion (Figures 1C,D). An MRI of the spine with contrast was performed demonstrating numerous new, subcentimeter enhancing osseous foci throughout her spine (Figure 2B). A CT of the neck, chest, abdomen, and pelvis and a thyroid US did not identify a potential site for primary disease. An F-18 FDG PET/CT scan performed at this time revealed that a hypermetabolic sclerotic lesion in the left iliac bone and a smaller sclerotic lesion in the right iliac bone without definite hypermetabolism (Figure 3).

A biopsy of a 0.5 cm right iliac lesion was attempted and demonstrated non-specific atypical cells that stained CD56+ on immunohistochemistry (IHC). The patient then underwent three lumbar punctures (LPs) with high opening pressures and elevated glucose but with negative cytology. Due to her high intracranial pressure, a brain biopsy was deferred at this time.

Two months later, the patient again presented to an emergency department following a generalized tonic-clonic seizure. A repeat LP revealed an increased opening pressure of 60 mm Hg and an MRI of the brain demonstrated the continued progression of her existing lesions in the parasagittal right frontal and parietal lobes with associated increasing infiltrative T2 signal. An MRI of the spine (see Figure 1) showed the progression of disease with an increase in size and number of multifocal enhancing osseous lesions of the spine (Figure 2C), MRI of the pelvis also showed new enhancing lesions. F18-FDG PET/CT was repeated now demonstrating FDG avidity of the increasing osseous lesions. All imaging was suggestive of a multifocal, neoplastic process but a slowly progressive atypical infection was not excluded.

Stereotactic biopsy of the right frontal lobe lesion was performed revealing an infiltrative, diffuse high-grade glioma with diffuse strong expression of p53. An H3F3A G34R mutation of specimen tissue was identified by pyrosequencing analysis. A bone marrow (BM) biopsy from the left anterior iliac crest was performed ~2 weeks following the brain biopsy. Evaluation of the bone marrow specimen revealed diffusely metastatic tumor with metastatic cells representing 30% of marrow cellularity within the sample. The immunohistochemical evaluation of the malignant cells revealed a diffuse strong expression for p53, ATRX expression, and focal weak expression for CD56. The profile of the malignant cells in the bone marrow were thought to be consistent with the recently diagnosed infiltrative brain glioma when compared together.

**Figure 4:**
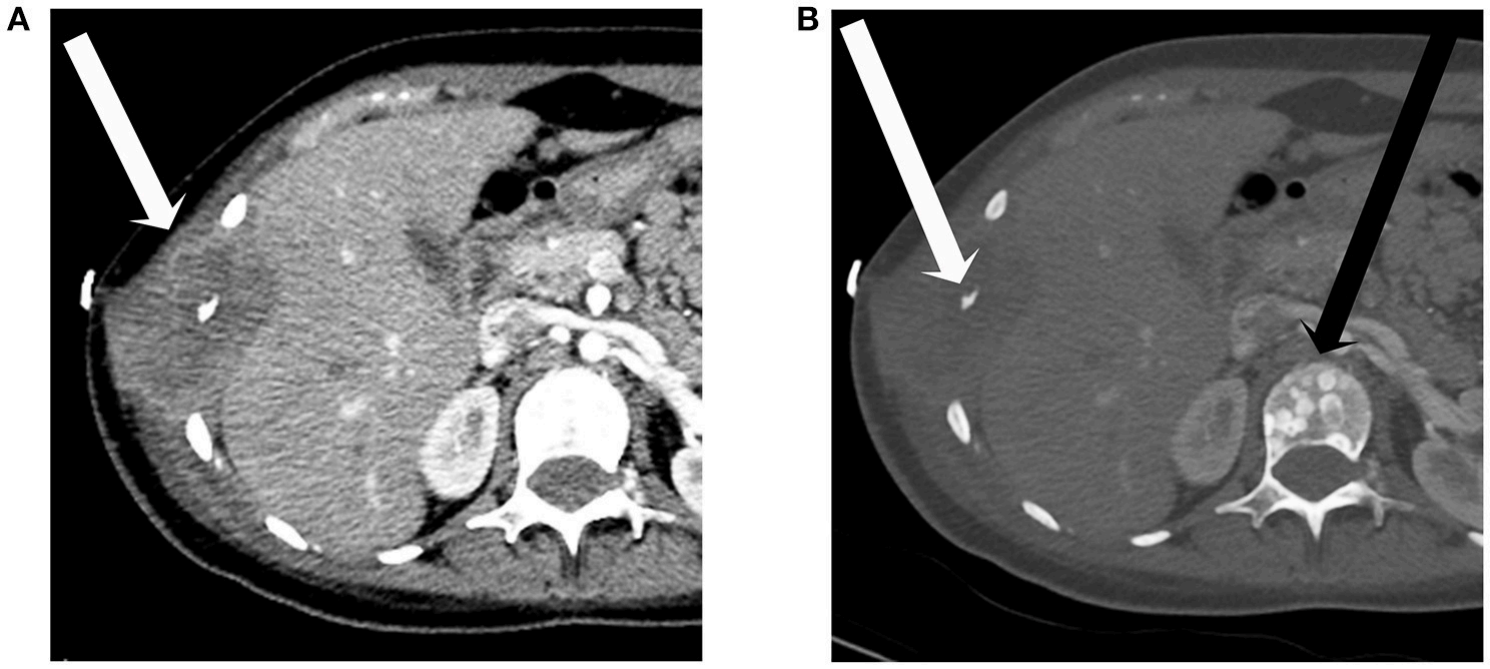
A 17-year-old female with H3F3A G34R mutated infiltrative glioma. An axial image from a CT of the chest with contrast in soft tissue window **(A)** reveals a destructive soft tissue lesion involving a lower right rib (arrow). The image in bone window **(B)** better demonstrates the rib destruction (white arrow) and also shows predominantly blastic lesions within the vertebra (black arrow).

During this admission the patient also underwent a lumbar puncture. Cerebrospinal fluid analysis revealed rare atypical cells thought to be compatible with the glioma. A CT of the chest with contrast performed also at this admission revealed the interval development of a large soft tissue mass in the right lower lateral chest wall centered around a rib (Figure 4). This was not present on the PET/CT study 9 months earlier. This mass was contiguous along the chest wall with underlying nodular pleural soft tissue lesions and a plural effusion. The patient underwent bilateral pleurocentesis with rare cells in the fluid staining positive for glial fibrillary acidic protein (GFAP).

**Figure 5:**
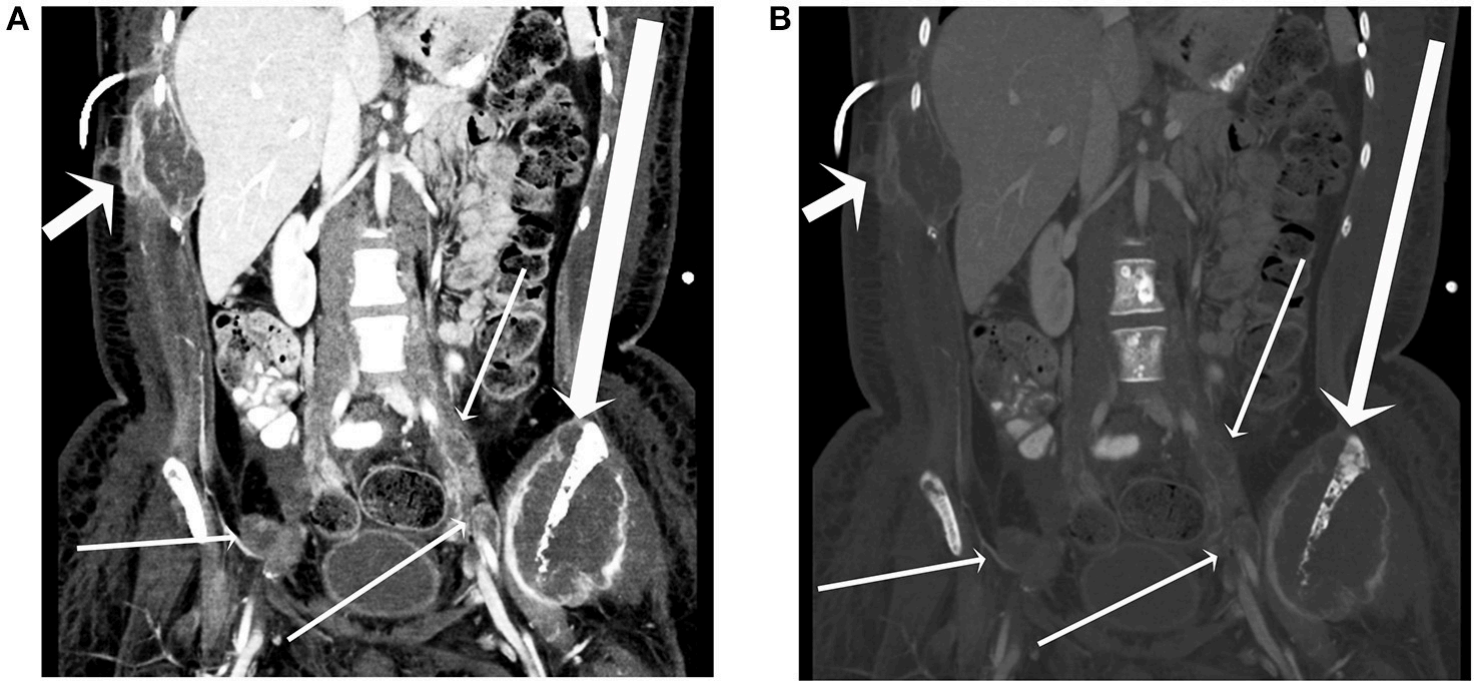
A 17-year-old female with H3F3A G34R mutated infiltrative glioma. A coronal image from a CT of the abdomen and pelvis with contrast in soft tissue window **(A)** and bone window **(B)** reveals a destructive lesion of the left iliac bone with large soft tissue component (long arrow), a destructive lesion centered around a right lower rib adjacent to a plural drainage catheter (short arrow), and iliac lymph node metastases (thin arrows).

The patient's metastatic disease continued to progress over the next 2 months with an CT of the abdomen and pelvis with contrast ~2 months after the last admission revealing diffuse osseous metastases throughout the bony pelvis and bilateral proximal femora (Figure 5). Extensive internal and external iliac adenopathy with encasement and compression of the iliac veins and inferior vena cava was also noted. As part of treatment planning, the dominant mass in the left pelvis underwent a CT-guided biopsy. Pathologic evaluation of the sample revealed malignant cells morphologically similar to the brain biopsy that stained positive for positive for GFAP and S100 protein.

## Discussion

Extraneural HGG metastasis is extremely rare, and the scarcity of this finding may lead to a delay in diagnosis and unnecessary diagnostic considerations. Here we describe the rapid development of widespread osseous metastatic disease from an infiltrative high-grade glioma with an H3F3A G34R mutation which is the first reported case from this genetic variant.

The extraneural spread of HGG most commonly occurs secondary to iatrogenic procedures. Examples include tumor implantation along biopsy tracts or seeding of the peritoneum through ventriculoperitoneal shunts, with at least 28 cases reported of pediatric primary brain tumors seeding to the peritoneum (7). In addition to surgery, other treatment modalities, such as radiotherapy (RT), can theoretically facilitate dissemination by damaging the surrounding healthy tissue, allowing tumors to invade past naturally-weakened barriers.

Obstacles to extraneural HGG metastasis include shortened patient lifespans, the presence of a thick dura around intracranial veins, and the inability of organ stroma to support and nurture the development of glial cells (4). Here, we describe a patient with a relatively prolonged life expectancy, whose disease rapidly progressed with metastatic disease to the axial skeleton spread before any surgical intervention, likely representing a rare instance of efficacious hematogenous or lymphatic spread outside the neuroaxis. Our patient underscores a more significant issue with our current lack of understanding of gliomagenesis and suggests unknown factors at play.

The presence of an H3F3A mutation was detected in two other documented pediatric HGG patients with bony metastases; however, our patient demonstrated a G34R mutation instead of K27M (6). Within H3F3A mutations, G34 mutations affect the development of highly-regulated transcription factors, such as distal-less homeobox 6 (DLX6), which encodes proteins responsible for forebrain development. Studies have explicitly linked the G34 gene to forebrain development and self-renewal as well as to upregulation of MYCN, a proto-oncogene regulating the first step in protein production. Defects in neurogenesis contribute to aggressive tumor biology, and multi-lobar extension of G34-mutated HGGs (including a gliomatosis cerebri pattern) has been reported in other retrospective reviews (7).

However, H3F3A mutations are not specific to HGGs and may occur in primary bone tumors, particularly giant cell tumors (GCTBs) and, to a much lesser extent, osteosarcomas (8–10). Direct sequencing of DNA from GCTB tissue reveals the presence of H3F3A mutations in up to 96% of cases. Sarcomas representing secondary malignant GCTBs also demonstrate a higher prevalence of the H3F3A mutation (9). High-grade “conventional-type” osteosarcomas exhibit a prevalence of 5.7% for H3F3A mutations. Although this estimate could be biased—owing to (1) misclassification of undifferentiated or *de novo* malignant GCTBs; (2) secondary transformation of malignant GCTBs; and (3) Denosumab-treatment effects leading GCTBs to mimic osteosarcomas (10). Immunohistochemical studies reveal GCTBs also express G34 mutations, particularly G34W aberrations (92%). In contrast, G34R mutations are relatively rare in GCTBs as well as other primary bone tumors (7).

Extraneural HGG metastasis likely has a complicated, multifactorial etiology that has yet to be delineated entirely. The identification of risk factors associated with this rare finding may result in earlier detection and treatment, potentially optimizing outcomes, since the presence of an H3F3A mutation in HGG is an important prognostic indicator. The mechanism of spread remains elusive and additional investigation is necessary to tease out its underlying pathogenesis.

## Ethics Statement

This study was carried out in accordance with the recommendations of The University of Texas MD Anderson Cancer Center. Case reports do not meet the institutional definition of research and are thus exempt from informed consent. No patient identifying information is included in this report.

## Author Contributions

AJ, MG-M, MC, and JJ contributed to the conception and design of the study. AJ wrote the first draft of the manuscript. MG-M, MC, and JJ wrote sections of the manuscript. All authors contributed to manuscript revision, read, and approved the submitted version.

### Conflict of Interest Statement

The authors declare that the research was conducted in the absence of any commercial or financial relationships that could be construed as a potential conflict of interest.
